# Water-soluble chlorophyll-binding proteins from *Brassica oleracea* allow for stable photobiocatalytic oxidation of cellulose by a lytic polysaccharide monooxygenase

**DOI:** 10.1186/s13068-020-01832-7

**Published:** 2020-11-30

**Authors:** N. Dodge, D. A. Russo, B. M. Blossom, R. K. Singh, B. van Oort, R. Croce, M. J. Bjerrum, P. E. Jensen

**Affiliations:** 1 Department of Food Science, University of Copenhagen, Rolighedsvej 26, 1958 Frederiksberg, Denmark; 2 Department of Bioorganic Analytics, Institute for Inorganic and Analytical Chemistry, Friedrich Schiller University Jena, Jena, Germany; 3 Department of Geosciences and Natural Resource Management, University of Copenhagen, Frederiksberg C, Denmark; 4 Department of Chemistry, University of Copenhagen, Copenhagen, Denmark; 5 Biophysics of Photosynthesis, Department of Physics and Astronomy, Faculty of Sciences, and LaserLaB Amsterdam, Vrije Universiteit Amsterdam, Amsterdam, The Netherlands

**Keywords:** Cellulose, Light-driven, Monooxygenases, Photobiocatalysis, Chlorophyll-binding protein

## Abstract

**Background:**

Lytic polysaccharide monooxygenases (LPMOs) are indispensable redox enzymes used in industry for the saccharification of plant biomass. LPMO-driven cellulose oxidation can be enhanced considerably through photobiocatalysis using chlorophyll derivatives and light. Water soluble chlorophyll binding proteins (WSCPs) make it is possible to stabilize and solubilize chlorophyll in aqueous solution, allowing for in vitro studies on photostability and ROS production. Here we aim to apply WSCP–Chl *a* as a photosensitizing complex for photobiocatalysis with the LPMO, *Tt*AA9.

**Results:**

We have in this study demonstrated how WSCP reconstituted with chlorophyll *a* (WSCP–Chl *a*) can create a stable photosensitizing complex which produces controlled amounts of H_2_O_2_ in the presence of ascorbic acid and light. WSCP–Chl *a* is highly reactive and allows for tightly controlled formation of H_2_O_2_ by regulating light intensity. *Tt*AA9 together with WSCP–Chl *a* shows increased cellulose oxidation under low light conditions, and the WSCP–Chl *a* complex remains stable after 24 h of light exposure. Additionally, the WSCP–Chl *a* complex demonstrates stability over a range of temperatures and pH conditions relevant for enzyme activity in industrial settings.

**Conclusion:**

With WSCP–Chl *a* as the photosensitizer, the need to replenish Chl is greatly reduced, enhancing the catalytic lifetime of light-driven LPMOs and increasing the efficiency of cellulose depolymerization. WSCP–Chl *a* allows for stable photobiocatalysis providing a sustainable solution for biomass processing.

## Background

Renewable and sustainable energy resources are necessary to sustain human consumption and decrease our reliance on fossil fuels [1]. Solutions for this can be found in nature where biological pathways exist that can convert sunlight into energy-rich biomass. Plant and algal biomass are renewable and can provide sustainable fuel alternatives including bioethanol, biodiesel and biogas [2]. Besides providing biomass, photosynthetic organisms have also inspired the development of photobiocatalysis, a biomimicry tool designed to speed up enzymatic reactions using light [3–5]. Photobiocatalysis has been shown to increase the activity of cytochrome P450s [6], methane monooxygenases (pMMO) [7] and lytic polysaccharide monooxygenases (LPMOs) [8–10].

LPMOs are soluble copper enzymes, found in fungi, bacteria and insects, among others, that aid in the natural decomposition and recycling of biomass [11]. Their copper active site is solvent exposed and coordinated by a histidine brace [12]. The flat binding surface and aromatic residues flanking the active site allow LPMOs to bind and cleave recalcitrant substrates such as chitin and cellulose [13, 14]. These enzymes are, therefore, used in current industrial enzyme cocktails to increase saccharification efficiency and glucose release [15]. LPMOs have proven particularly useful at higher substrate loadings by synergistically enhancing the hydrolytic activity of cellulases [16, 17].

For their catalytic cycle, LPMOs require an external reductant [18] and one of two cosubstrates, molecular oxygen (O_2_) or hydrogen peroxide (H_2_O_2_) [19]. The cosubstrates interact with a reduced copper active site forming a reactive intermediate which can then oxidize the substrate. Recent studies have demonstrated significantly higher product yields when H_2_O_2_ is involved in LPMO catalysis [20, 21]. However, the amount of H_2_O_2_ has to be controlled as high concentrations have been shown to be detrimental to LPMO activity [19].

The first report of light-driven LPMOs by Cannella et al. demonstrated that LPMOs can also be light-driven [8]. This work proposed that the enhanced light-driven LPMO activity is due to a photoactivated electron transfer from a photosynthetic pigment directly to the LPMO. However, recent evidence indicates thats the formation of H_2_O_2_ by a photosensitizer is involved in the acceleration of light-driven LPMO catalysis [9, 10]. Regardless of the exact mechanism, light-driven reactions have the potential to provide more powerful, faster and, thus, ‘greener’ redox reactions [22].

Although photobiocatalysis is a relatively new field, photosensitizers have been used in a variety of applications, and tend to follow two main photodynamic mechanisms in the presence of oxygen [23]. Type I is electron transfer, where excited sensitizers can reduce oxygen resulting in superoxide (O_2_^-^), whereas Type II involves energy transfer from the photosensitizer to oxygen producing singlet oxygen (^1^O_2_) [24]. Natural photosensitizers are made up of porphyrin ring-structured molecules, such as in chlorophyll (Chl) and its derivatives. Chl is one of the Nature’s most abundant and powerful photosensitizers: however, the utilization of Chl in an industrial setting remains a challenge. Although chlorophyll molecules are quite stable within their native environment, in protein complexes of the thylakoid membrane, in an aqueous solution, Chl molecules are highly insoluble and become more prone to photooxidation.

One way to stabilize Chl in solution is through reconstitution with water-soluble chlorophyll-binding proteins (WSCPs). These soluble proteins form tetrameric complexes with Chl and have been shown to considerably increase photostability. Typically, Chl-binding proteins are hydrophobic, membrane bound complexes, such as reaction centers and light-harvesting complexes involved in photosynthesis [25]. Chl binding proteins typically protect Chl from photooxidation with the presence of carotenoids. WSCPs, however, do not contain carotenoids, but have been shown to have a similar photostabilizing effect on Chl. Although their biological function remains largely unknown, WSCP is the only known soluble Chl-binding protein found in higher plants [26]. WSCP complexes have shown no involvement in photosynthesis; however, the cytosolic formation of reactive oxygen species could indicate a role in protection against pathogen attack [27]. Furthermore, these proteins have been localized in the endoplasmic reticulum bodies, only found in *Brassicaceae* plants, and thought to be involved in the stress response and injury [28]. It is believed that WSCPs are able to stabilize Chl by creating a physical barrier, shielding the phytyl chain and magnesium ion from the surrounding solution and oxidative damage [29].

It has previously been shown that WSCP–Chl *a* complexes remain functional after prolonged incubation at high temperatures, as well as at extreme pH values, providing potential for industrial application [26]. Therefore, in this work, we propose to utilize WSCPs to bind Chl and prolong photosensitizer lifetime and, consequentially, productivity of a light-driven LPMO system. To this end, a 22 kDa WSCP was reconstituted with Chl *a* resulting in a tetrameric WSCP–Chl *a* complex which was tested as a photosensitizer for light-driven activity of the *Tt*AA9 LPMO from *Thielavia terrestris*. The stability of the WSCP–Chl *a* complex, and its ability to drive the LPMO, was tested under various light, temperature and pH conditions to demonstrate the robustness of this system.

## Results

### Stability of WSCP–Chl *a* versus free Chl *a*

One of the central aims of photobiocatalysis is to use light to drive enzymes that catalyze reactions of interest such as the degradation of recalcitrant substrates like cellulose. For application of light-driven systems, the lifetime of the photosensitizer is, therefore, vital to prolong the catalytic lifetime.

The photostability of free Chl *a* and Chl *a* bound to the WSCP (WSCP–Chl *a*) was measured over time in an LPMO light-driven system which includes *Tt*AA9 and a reductant (ascorbic acid, Asc). Photostability is in this context defined as the loss of Chl *a* fluorescence over time relative to initial fluorescence (*F*/*F*_0_).

**Fig. 1:**
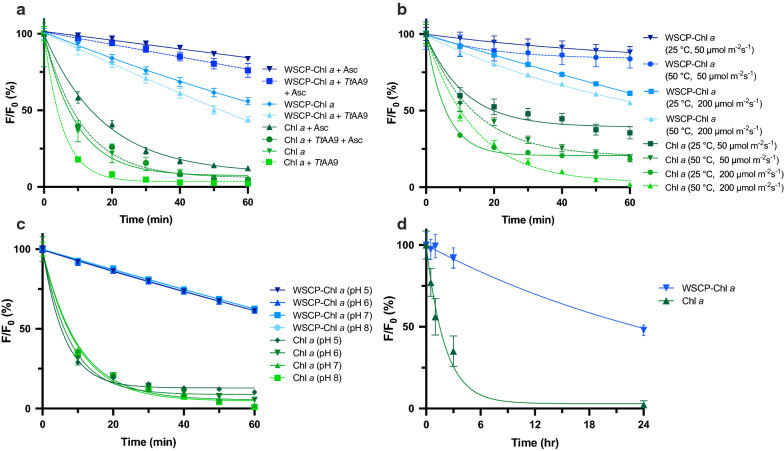
Photostability of WSCP–Chl *a* and free Chl *a*. Fluorescence measured every 10 min (F) is divided by starting fluorescence (F_0_) to show the loss over time. Blue lines represent WSCP–Chl *a* complexes (2.6 µM) and green lines represent free Chl *a* (2.6 µM) in 0.03% ß-DM. Each time point is the average of three independent experiments shown with standard error of the mean (SEM). All curves were fitted using an exponential one phase decay model (*R*^*2*^ > 0.97). **a** Comparing the effect of LPMO assay components on the stability of WSCP–Chl *a* and free Chl *a*. Assay components were in the following concentrations: Asc (1 mM), *Tt*AA9 (0.035 mg mL^−1^), 50 mM sodium phosphate buffer (pH 6.3). Conditions: 25 °C and 500 µmol m^−2^ s^−1^ white light (4000 K). **b** WSCP–Chl *a* and Chl *a* under different light and temperature conditions (25 °C/50 °C; 50/200 µmol m^−2^ s^−1^) containing pigments (2.6 µM) and 50 mM sodium phosphate buffer (pH 6.3). **c** Photostability of WSCP–Chl *a* and Chl *a* at different pH. Assay composed of pigments (2.6 µM) and 50 mM potassium phosphate buffer (pH 5-8). Conditions: 25 °C and 500 µmol m^−2^ s^−1^. **d** 24-h photostability assay performed at 50 µmol m^−2^ s^−1^ with pigments (2.6 µM) and 50 mM sodium phosphate buffer (pH 6.3). All assays were performed with cool white LEDs (4000 K) spectrum

As expected, we observed that, in the light-driven LPMO system, the WSCP–Chl *a* complex is more stable than free Chl *a* in all conditions (Fig. 1a). When combined with *Tt*AA9 and Asc, WSCP–Chl *a* showed 76 ± 4% fluorescence after 1 h compared to Chl *a* where only 5 ± 0.2% remained. In the partial assay systems, ascorbic acid enhances the photostability of both the WSCP–Chl *a* complex and Chl *a*, whereas, the presence of the *Tt*AA9 decreases the apparent photostability. However, when combining the *Tt*AA9 and Asc the negative effects caused by the enzyme seem to be counteracted for both WSCP–Chl *a* and Chl *a*. The final fluorescence ratio (F/F_0_ at 60 min) was analyzed with single factor ANOVA. All WSCP–Chl *a* samples were significantly different from each other (p < 0.001).

### Effect of light and temperature on the stability of WSCP–Chl *a* versus free Chl *a*

For application of photobiocatalytic LPMO reactions, the photosensitizer should ideally be stable under a broad range of temperatures. For example, several fungal LPMOs (AA9) have been shown to have the highest activity levels at temperatures ranging between 40 and 50 °C [30]. Therefore, photostability was tested at 25 and 50 °C. Together with temperature, light intensity was also varied to investigate which of the two factors has a larger influence on the photostability of the complex. Both photosensitizers were subjected to 50 and 200 µmol m^−2^ s^−1^ for 1 h at 25 and 50 °C (Fig. 1b). As expected, lower light conditions (50 µmol m^−2^ s^−1^) were beneficial for both WSCP–Chl *a* and free Chl *a*. When the light was increased to 200 µmol m^−2^ s^−1^, a considerable loss in photostability was observed. The rise in temperature causes an extra 15% loss of fluorescence in free Chl *a* compared to only 5% in WSCP–Chl *a* complex.

### Effect of pH on stability of WSCP–Chl *a* versus free Chl *a*

Many enzymatic reactions require rather acidic or basic environments. For example, LPMO containing enzyme cocktails have been shown to achieve a maximum depolymerization at pH 5 [31, 32]. Therefore, it is important that a photosensitizer remains stable across a broad range of pH-values. To determine the pH stability of the WSCP–Chl *a* complex and free Chl *a*, both were incubated in different buffers, ranging from pH 5–8, and their photostability was measured over time (Fig. 1c).

The photostability of WSCP–Chl *a* is unaffected by the changes in pH as there is no significant difference between all four samples with single factor ANOVA (p > 0.05). The stability of free Chl *a* is expected to be favored by high pH as these pigments are known to lose their central Mg ion in acidic conditions [33, 34]. Although this effect is not seen under our experimental conditions, Chl *a* remains unstable with between 4 and 12% fluorescence remaining after 60 min under all conditions. WSCP–Chl *a* retained between 61 and 63% at pH 5–8.

### Photostability of WSCP–Chl *a* and Chl *a* after 24 h

A 24-hour assay was done at low light (50 µmol m^−2^ s^−1^) to demonstrate the long-term stability of WSCP–Chl *a* (Fig. 1d). One phase decay model was used to approximate the half-life of both pigments with a confidence interval over 95%. Chl *a* shows a half-life of approximately 1.5 h while WSCP–Chl *a* is estimated at approximately 24 h.

### Effect of light intensity on H_2_O_2_ production

**Fig. 2:**
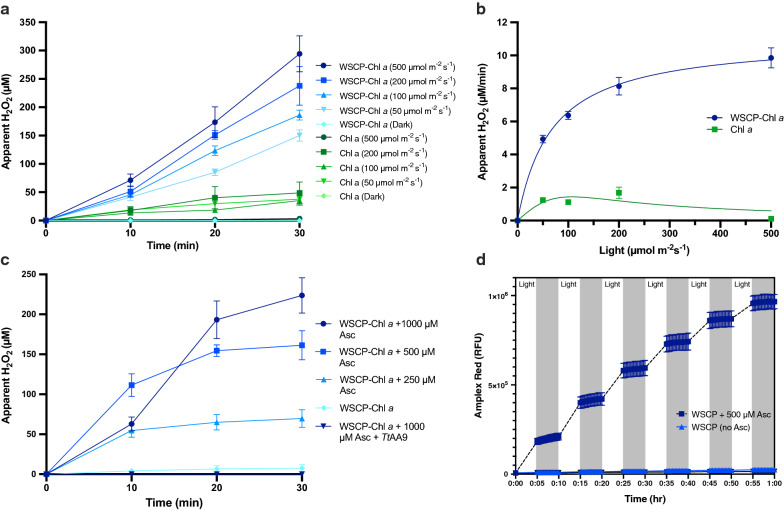
Light dependent H_2_O_2_ formation of WSCP–Chl *a* under different conditions. Using an Ampliflu™ assay, H_2_O_2_ accumulation was measured under different conditions. Blue lines represent WSCP–Chl *a* complexes and green lines represent unbound Chl *a* in 0.03% β-DM. Each point is the average of three independent experiments and the corresponding SEM. Light assay components were in the following concentrations: pigments (2.6 µM), and 50 mM sodium phosphate buffer (pH 6.3). **a** Five different light intensities were tested: 500, 200, 100, 50, and 0 (dark control) µmol m^−2^ s^−1^ cool white light (4000 K) spectrum. Assays composed of: pigments (2.6 µM), Asc (1 mM), and 50 mM sodium phosphate buffer (pH 6.3). **b** Rate constants of H_2_O_2_ formation at different light intensities calculated from each curve under the 5 light intensities (0, 50, 100, 200, 500 µmol m^−2^ s^−1^) for both WSCP–Chl *a* and unbound Chl *a*. **c** Five different conditions were tested with WSCP–Chl *a*: 1000, 500, 250, 0 µM Asc and 1000 µM Asc + *Tt*AA9 (0.035 mg mL^−1^). All samples contained CNF (0.25% w/v). Light intensity was set to 500 µmol m^−2^s^−1^. The final H_2_O_2_ concentration (30 min) was analyzed with single factor ANOVA for all WSCP–Chl *a* samples (not including the LPMO control). **d** Light/dark accumulation of H_2_O_2_ with WSCP–Chl *a* in the presence and absence of Asc, alternating between 5 min in the 50 µmol m^−2^ s^−1^ followed by 5 min of fluorescence measurements in the dark

In light of the recent publications suggesting H_2_O_2_ is a key factor in light-driven LPMOs [9] we proceeded to investigate the light-driven formation/generation of H_2_O_2_ from WSCP–Chl *a* and free Chl *a* under varying light intensities (0, 50, 100, 200, and 500 µmol m^−2^ s^−1^) (Fig. 2a). With WSCP–Chl *a*, higher light intensities lead to a faster rate of H_2_O_2_ formation, as measured by the Ampliflu™ assay, with a maximum value of 298 µM after 30 min in 500 µmol m^−2^ s^−1^ light. The highest H_2_O_2_ formation seen in free Chl *a* is at 200 µmol m^−2^ s^−1^ with a total of 60 µM H_2_O_2_ after 30 min. In the absence of light, no formation of H_2_O_2_ is observed. Interestingly, Chl *a* exposed to a light intensity of 500 µmol m^−2^ s^−1^ also showed greatly reduced formation of H_2_O_2_ compared to the same experiment with light intensity of 200 µmol m^−2^ s^−1^. This is likely a result of rapid photobleaching of Chl *a* in the first minutes of the reaction (Fig. 2a). To establish the correlation between light intensity and H_2_O_2_, the time traces in Fig. 2a were each fitted with a linear function. The resulting slopes correspond to the rate of H_2_O_2_ measured per minute (Fig. 2b).

### Effect of reductant concentration and light on H_2_O_2_ formation

After having determined the correlation between light intensity and H_2_O_2_ formation, we investigated the effects of the reductant (Asc) concentration on H_2_O_2_ formation. The assay was set up with WSCP–Chl *a* and four different concentrations of Asc (0, 250, 500, and 1000 µM) (Fig. 2c). Higher concentrations of Asc led to greater endpoint H_2_O_2_ formation, however, 1000 µM Asc forms similar concentrations of H_2_O_2_ as 250 µM Asc after 10 min. H_2_O_2_ levels were below the detection limit in the absence of Asc, indicating the necessity of reductant in the light-driven mechanism. With 1000 µM Asc, the addition of *Tt*AA9 reduces the amount of detected H_2_O_2_ to that in the absence of Asc, suggesting that either *Tt*AA9 prevents the formation of H_2_O_2_, or, more likely, that *Tt*AA9 degrades H_2_O_2_ that is formed before it can react with Ampliflu™. To demonstrate the tight control of H_2_O_2_ production by WSCP–Chl *a,* a light–dark alternating assay was performed. In this assay, WSCP–Chl *a* was placed in 50 µmol m^−2^ s^−1^ for 5 min followed by 5 min of fluorescence measurements in the dark (Fig. 2d). This assay clearly demonstrates the light-dependence of H_2_O_2_ production by WSCP–Chl *a.* Once again, this assay also shows the importance of Asc in the system for H_2_O_2_ production.

### *Light*-*driven Tt*AA9 *assays*

**Fig. 3:**
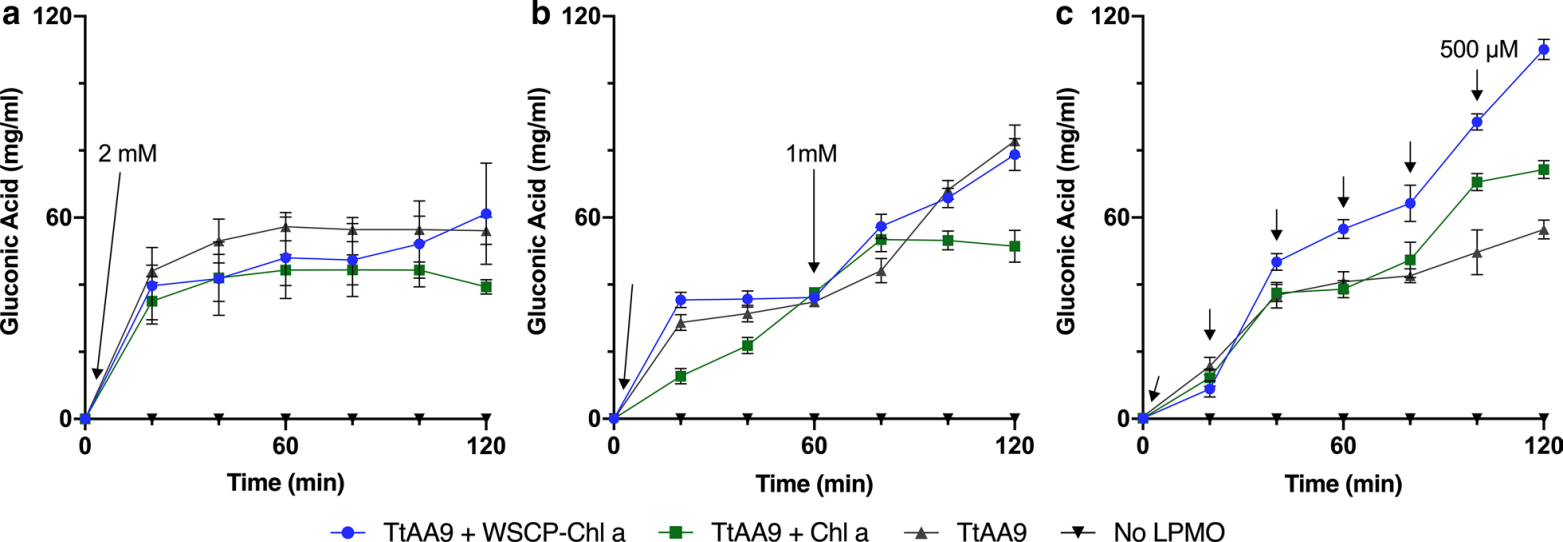
Gluconic acid determination after light driven *Tt*AA9 time course assays under various Asc feed rates. All three assays were performed at 100 µmol m^−2^ s^−1^ cool white LED (4000 K) spectrum at 25 °C in 50 mM sodium phosphate buffer (pH 6.3), with CNF (0.25% w/v) and either *Tt*AA9 and WSCP–Chl *a*, *Tt*AA9 and Chl *a*, *Tt*AA9 without pigment, or WSCP–Chl *a* as a no enzyme control. Asc feed rates were: **a** 2 mM Asc, **b** 1 mM Asc/hour, or **c** 500 µM every 20 min. Curves are averages of three independent experiments and the SEM of these experiments is shown. Single factor ANOVA was done on the final gluconic acid concentration (120 min) between *Tt*AA9 w/ and w/o WSCP–Chl *a*. Dark and control reactions can be found in Additional file 1: Fig. S6

To assess whether the higher photostability of the WSCP–Chl *a* would lead to higher *Tt*AA9 product formation, light-driven assays were performed with *Tt*AA9 using varying concentrations of reductant. Since high concentrations of H_2_O_2_ can be detrimental to LPMO activity [19, 20, 35], and the WSCP was shown to be more stable at lower light intensities (Fig. 1b), the light intensity was reduced to 100 µmol m^−2^ s^−1^ for *Tt*AA9 experiments. Subsequently, the optimization process was focused on the “feed rate” of Asc to control the H_2_O_2_ production in the assays. The feed rate is defined as the concentration (mM) of Asc added at certain time intervals (min). It is difficult to determine the necessary reductant concentration since there are many factors involved. To demonstrate the importance of reductant concentrations, three assays with varying Asc feed rates were set up: 2 mM Asc (Fig. 3a), 1 mM Asc every 60 min (Fig. 3b), and 500 µM every 20 min (Fig. 3c), with gluconic acid concentrations measured every 20 min for 2 h.

In Fig. 3a, in the presence of 2 mM Asc, all samples show high productivity in the first 20 min, after which productivity halts. Final concentrations were 61, 39, and 56 (mg L^−1^) for samples WSCP–Chl *a*, free Chl *a*, and no pigment, respectively. Upon halving the concentration of Asc, and adding it every hour (1 mM/h), there is a noticeable increase in productivity of all samples (Fig. 3b); however, there is still no significant increase in *Tt*AA9 productivity upon the addition of WSCP–Chl *a*. In the final assay (Fig. 3c), the concentration of Asc was changed to 500 µM, added every 20 min, which led to significantly increased productivity (p < 0.05; determined for t = 120 min) for both assays containing photosensitizers. *Tt*AA9 with WSCP–Chl *a* resulted in a final gluconic acid concentration of 110 mg L^−1^. Chl *a* also boosted *Tt*AA9 productivity significantly with 75 mg L^−1^ compared to *Tt*AA9 alone at 59 mg L^−1^.

**Fig. 4:**
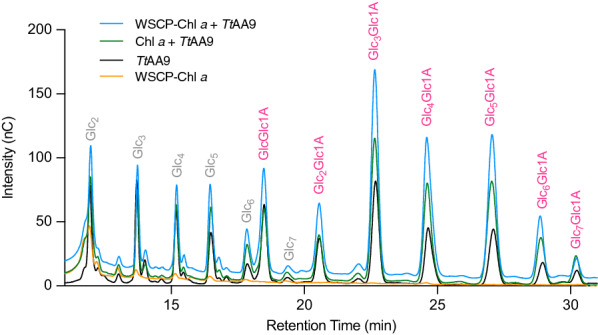
HPAEC Chromatograms of *Tt*AA9 with WSCP–Chl *a*, *Tt*AA9 with Chl *a*, *Tt*AA9 without any pigments, and WSCP–Chl *a* control without *Tt*AA9. These were taken after 3 h at 50 µmol m^−2^ s^−1^ and 50 °C with 50 mM potassium phosphate buffer (pH 6), with CNF (0.25% w/v) and 500 µM Asc/h. Peaks were assigned based on standards: cellobiose (Glc_2_), cellotriose (Glc_3_), cellotetraose (Glc_4_), cellopentaose (Glc_5_), cellohexaose (Glc_6_), and celloheptaose (Glc_7_). C1-oxidized oligosaccharides (pink) are cellobionic acid (GlcGlc1A), cellotrionic acid (Glc_2_Glc1A), cellotetraonic acid (Glc_3_Glc1A), cellopentaoinic acid (Glc_4_Glc1A), cellohexaoinic acid (Glc_5_Glc1A), celloeptaonic acid (Glc_6_Glc1A), and cellooctaonic acid (Glc_7_Glc1A)

High-Performance Anion-Exchange Chromatography (HPAEC) was performed to confirm the results of the gluconic acid measurements. The slightly acidic nature of carbohydrates allows for highly selective separations using anion exchange at high pH. The C1-oxidized products are easily characterized as seven distinct singular peaks (Fig. 4). For this study, the chromatograms can be used to compare the relative signal intensities of the oxidized products in the different samples. *Tt*AA9 productivity after 3 h with WSCP–Chl *a* shows a max signal intensity of 168.8 (nC) at 22 min corresponding to cellotetraonic acid (Glc_3_Glc1A). *Tt*AA9 + Chl *a* and *Tt*AA9 on its own also demonstrate max intensities with Glc_3_Glc1A at 115.31 and 81.2 (nC), respectively. A control containing WSCP–Chl *a* with no *Tt*AA9 shows no C1 oxidations peaks the visible cellobiose (Glc_2_) is background from the substrate. The area under the C1 oxidation peaks was used to estimate the photobiocatalytic enhancement. The area for *Tt*AA9 + Chl *a* was 1.88× than TtAA9 alone, while TtAA9 + WSCP–Chl *a* was 3.4× greater compared to *Tt*AA9 alone.

## Discussion

Most biological pigments, and in particular chlorophyll, are prone to rapid photooxidation if exposed to light outside of their natural environment. Due to this, the use of biological pigments in photobiocatalysis is still limited. Therefore, for future application, it is of interest to describe novel pigment systems capable of withstanding the, potentially, harsh biomanufacturing conditions. The work reported here presents WSCP–Chl *a* as a possible candidate for industrial application of chlorophyll-based photosensitizers. Through the controlled light-induced formation of H_2_O_2_, it was possible to adjust conditions to obtain increased *Tt*AA9 productivity. Overall, *Tt*AA9 productivity was enhanced over threefold and confirmed using various detection methods.

### Photostability assays

The WSCP–Chl *a* complexes were tested under various conditions to confirm the photooxidative protection properties of the WSCP [25, 26]. The tested conditions included individual and combinations of assay components including *Tt*AA9 and Asc. Most notably we observed a decreased photostability in the WSCP–Chl *a* complex as well as the free Chl *a* caused by the presence of *Tt*AA9 (Fig. 1a). However, Asc appears to counteract this negative side effect most likely due to its antioxidant properties. This was seen by the restoration of photostability in both WSCP–Chl *a* and free Chl *a* when *Tt*AA9 and Asc are combined.

Light and temperature effects were also tested on both WSCP–Chl *a* and Chl *a.* Overall improvement of photostability is seen in all conditions for both WSCP–Chl *a* and Chl *a* since the light intensity is lowered considerably. At 500 µmol m^−2^s^−1^, with pigment alone, WSCP–Chl *a* retained 56 ± 1.8% fluorescence after 60 min, compared to 61.2 ± 1.1% and 87.9 ± 4% at 200 and 50 µmol m^−2^s^−1^, respectively. Chl *a* shows some improvement from 4.6 ± 1.3% at 500 µmol m^−2^s^−1^, to 29.1 ± 0.6% and 43.9 ± 3.5% at 200 and 50 µmol m^−2^s^−1^, respectively. As expected, light intensity has a bigger influence on photostability than temperature (Fig. 1b). Temperature is important for the activity of the LPMOs as most assays are performed at 45–50 °C [36]. The loss of 4.5% photostability is minimal at 50 °C when considering the overall goal is increased productivity of *Tt*AA9. Another important factor for LPMO activity is pH. LPMO assays are generally performed around pH 5–7 [36]. The WSCP–Chl *a* showed no change in photostability between pH 5 and 8 (Fig. 1c). To demonstrate the long-term stability of WSCP–Chl *a*, a 24 h assay was run at 50 µmol m^−2^s^−1^ (Fig. 1d). Lifetimes were estimated using a one phase decay model. WSCP is estimated to increase pigment half-life 16-fold from 1.43 h. to 23.34 with WSCP–Chl *a*.

### H_2_O_2_ assays

WSCPs bound to Chl *a* have been shown to produce large quantities of ^1^O_2_ when exposed to light. This was confirmed by fluorescence detection using Singlet Oxygen Sensor Green (SOSG) (Additional file 1: Fig. S1), and was performed according to Agostini et al. [29]. It has furthermore been demonstrated that Asc reacts readily with ^1^O_2_ to form H_2_O_2_ [37]. In the context of LPMOs, H_2_O_2_ has been shown to increase LPMO activity in several instances [9, 18, 38]. That being said, H_2_O_2_-driven LPMO catalysis has two sides. To increase activity, the concentration of H_2_O_2_ has to be optimal for the LPMO to function. High concentrations of H_2_O_2_ can lead to self-inactivation of reduced non-substrate-bound LPMOs [21]. In an attempt to determine the rate of H_2_O_2_ produced by WSCP–Chl *a* with Asc, several assays were performed under varying light conditions. Higher light intensities led to greater H_2_O_2_ formation as seen in Fig. 2a. As long as there is Asc present in the reaction, then formation of H_2_O_2_ is expected to increase continuously until the pigments are degraded. However, the rate of H_2_O_2_ generation does not increase linearly with light intensity (Fig. 2b). These results can be used to adjust the light intensity for an estimated production of H_2_O_2_ µM/min. However, the exact value is difficult to estimate due to the fact that Asc is also involved in scavenging H_2_O_2_ [39]. As seen in Fig. 2c, higher concentrations of Asc do not necessarily lead to more H_2_O_2_ initially. There appears to be competition between formation and scavenging reactions by Asc. H_2_O_2_ is able to oxidize Asc as well as resulting oxidation products such as dehydroascorbic acid and 2,3-diketoguloric acid [40]. Due to these unavoidable side reactions, precise quantification of H_2_O_2_ is uncertain and the resulting measurements are therefore referred to as ‘apparent’ (Fig. 2).

Substrate (CNF) was also present in Fig. 2c, which could explain the variations seen in this experiment and Fig. 2a. CNF at 0.25% (w/v) is a viscous substrate which interferes with homogeneity, and thus reproducibility, causing slight deviations in the results. Furthermore, CNF is known to cause light scattering which would account for the overall lower levels of apparent H_2_O_2_ [10]. Despite these competing reactions and interferences, H_2_O_2_ formation remains tightly controlled under light and dark incubation as seen in Fig. 2d. This reaction is unmistakably dependent on Asc and light. LPMOs have been shown to consume H_2_O_2_ in the presence of substrate [19], which is also demonstrated in Fig. 2c. This experiment which was performed with the substrate (CNF) contained a control reaction *Tt*AA9, resulting in negligible amounts of H_2_O_2_ throughout the course of the experiment (Fig. 2c). This supports the hypothesis that reduced, substrate-bound LPMOs possess peroxygenase activity [19] and that *Tt*AA9 is able to consume H_2_O_2_ produced by WSCP–Chl *a*.

### *Light*-*driven Tt*AA9 *assays*

Two different methods were used for determining *Tt*AA9 productivity: gluconic acid estimation and detection of C1 oxidized products by HPAEC–PAD. Gluconic acid assays were used to quantify C1-oxidations of *Tt*AA9 as this is the primary function of Type I LPMOs. Three time courses were performed with reductant added at different intervals [38] to demonstrate the influence of Asc on *Tt*AA9 productivity (Fig. 3a–c). By controlling reductant concentration, we can limit the accumulation of H_2_O_2_ at a given time. Previous reports have shown that while low concentrations of H_2_O_2_ are beneficial, high concentrations can be detrimental to LPMOs and lead to enzyme inactivation. Lowering the concentrations of H_2_O_2_ and Asc and adding with higher frequency have previously been shown to be beneficial to LPMO productivity [21].

Based on Fig. 2b, it can be estimated that, with 2 mM Asc there is at least 200 µM H_2_O_2_ produced by WSCP–Chl *a* after 20 min (Fig. 3a). In Fig. 3b we estimated a concentration of 123 µM H_2_O_2_ after 20 min with 1 mM Asc. This could potentially explain why some light-driven samples appear to hinder *Tt*AA9 productivity. Concentrations over 100 µM H_2_O_2_ have been shown to lead to enzyme inactivation [19, 38]. *Tt*AA9 catalysis seems to be primarily Asc-driven in Fig. 3a and 3b and that light does not provide much benefit in the presence of large amounts of Asc. After optimization, the effect of light is evident with nearly twice the product formation upon the addition of WSCP–Chl *a* (Fig. 3c). Figure 3c also demonstrates that the importance of a photosensitizer for light-driven assays. Based on the photostability assays (Fig. 1), Chl *a,* is almost entirely inactive after 60 min, thus diminishing any added value during longer reactions.

The signal intensities from the HPAEC confirm LPMO substrate oxidation with similar result as Fig. 3c. After three hours WSCP–Chl *a* with *Tt*AA9 is the most active, followed by Chl *a* with *Tt*AA9 and then *Tt*AA9 alone (Fig. 4). Similar HPAEC chromatograms were seen for *Tt*AA9 with chlorophyllin and light [8]. Ultimately, all components of the reaction are important and light can only be beneficial given that the other components are balanced correctly.

### Light–driven formation of H_2_O_2_ with WSCP–Chl *a*

Cannella et al. was the first to show light-driven activation of LPMO in the presence of pigments and ascorbic acid [8]. They hypothesized that upon light-excitation, pigments would become excited and then transfer an electron directly to the LPMO [8]. The reductant, Asc or lignin, would be responsible for replenishing the donated electrons in the pigments, allowing for further excitation and electron transfer. A recent publication by Bissaro et al. [9], also utilized chlorophyllin to drive an LPMO resulting in large quantities of H_2_O_2_ and superoxide (O_2_^−^) which would explain the light-driven enhancement by photosynthetic pigments.

**Fig. 5:**
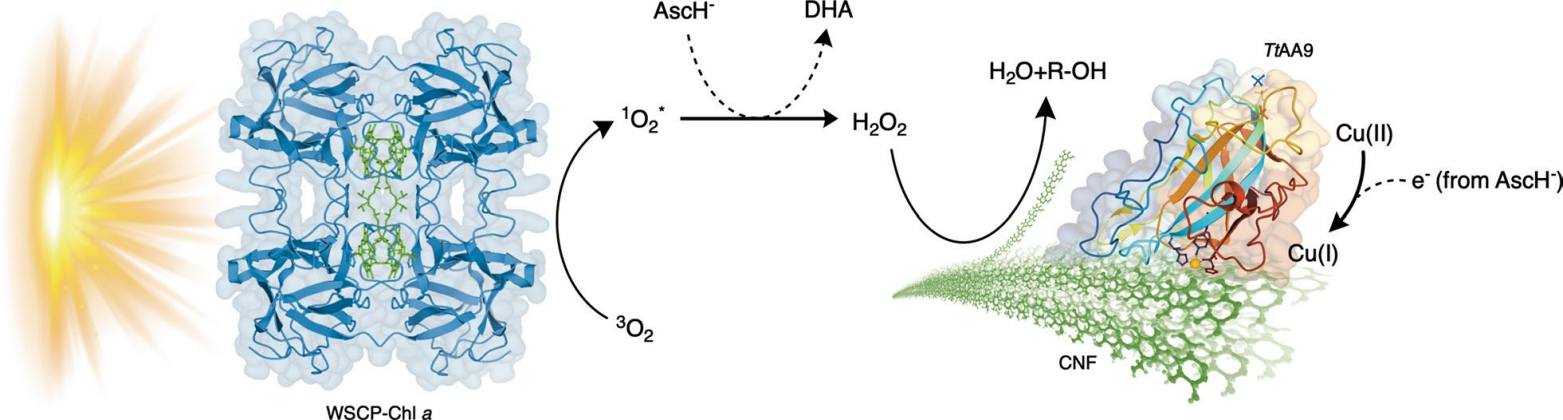
Proposed ROS-mediated light-driven enhancement of *Tt*AA9 with WSCP–Chl *a*. Triplet excited Chl *a* transfer excitation energy to ^3^O_2_ forming ^1^O_2_ upon light exposure. In the presence of AscH^-^, ^1^O_2_ is reduced to H_2_O_2_ which can be used by a reduced Cu(I)-*Tt*AA9 for hydrolysis of CNF. *Tt*AA9 (PDB: 3EJA), WSCP1 tetramer reconstituted with Chl *a*. (PDB: 6S2Z)

Data from this study also confirm that H_2_O_2_ is produced in the presence of Asc, pigment and light and is therefore likely involved in the catalytic enhancement of *Tt*AA9 . In this study and under these conditions, there was no evidence to suggest that O_2_^-^ is involved in the light driven enhancement of *Tt*AA9. Superoxide dismutase (SOD) was added to WSCP–Chl *a* and TtAA9 but showed no significant enhanced effect on the productivity of *Tt*AA9 (Additional file 1: Fig. S2). There was, however, evidence to confirm the abundant production of ^1^O_2_ by WSCP–Chl *a* (Additional file 1: Fig. S1) as demonstrated by Agostini et al. [29]. Based on this information, a proposed reaction scheme from WSCP–Chl *a* can be seen in Fig. 5.

It is possible that the light intensity and pigment composition could play a role in type of ROS formed and further studies should be done to determine the exact mechanism behind this. The production of O_2_^-^ could allow for Bissaro et al. [9] to drive an LPMO without Asc, however, this was with higher light intensities and different pigments. Chlorophyllin is an undefined mix of water-soluble chlorophyll derivatives [41] that may, in fact, produce O_2_^−^. However, as explained above this was most likely not the case for WSCP–Chl *a* as used here. Due to the photostability of WSCP–Chl *a*, low light (50 µmol m^−2^s^−1^) and very little WSCP–Chl *a* (2.6 µM) are needed to produce enough H_2_O_2_ for successful photocatalysis of *Tt*AA9. For comparison, 50–100 µmol m^−2^s^−1^ is measured as instantaneous photosynthetic photon flux density (PPFD) on a cloudy winter day in Northern Europe [42]. This proof of concept study invites further investigation into the applications of WSCP–Chl *a* with other H_2_O_2_-driven enzymes. LPMOs can also vary in their substrate specificity and these results should therefore be confirmed with other types of LPMOs.

## Conclusion

In this study, a recombinant WSCP from *Brassica oleracea var. botrytis* (WSCP1), was reconstituted with Chl *a* to form a stable tetrameric complex (WSCP–Chl *a*). WSCP–Chl *a* was then tested for photostability under various conditions. These conditions were designed to test the use of this complex as a new photosensitizer for the emerging field of photobiocatalysis. The complex displayed superior stability under high light conditions, as well as varying pH and in combination with enzymatic assay components. It was further shown that in combination with Asc, WSCP–Chl *a* formed H_2_O_2_ when exposed to light. The H_2_O_2_ formation was dependent on both the reductant (Asc) concentration and the light intensity. To test the use of this application of this new photosensitizer, WSCP–Chl *a* was combined with the C1 oxidizing *Tt*AA9 to enhance hydrolytic activity upon light exposure. With the recent discovery of LPMO peroxygenase activity, it is proposed, that WSCP–Chl *a* can produce steady amounts of H_2_O_2_ upon light exposure which can in turn enhance LPMO activity. Light-driven assays yielded a two- to threefold increase in productivity which was confirmed using both gluconic acid determination and HPAEC chromatograms. Based on the data provided in this study, it is believed that WSCP–Chl *a* is promising addition to the field of photobiocatalysis.

## Methods

### Construct design and plasmid generation

The amino acid (AA) sequence of the mature WSCP1 protein (AA 20–218) from *Brassica oleracea var. botrytis* (UniprotKB: Q7GDB3) was custom synthesized by GenScript (USA) and inserted into the TOPO^®^ cloning site of the pET151/D-TOPO^®^ vector (Invitrogen) by overlap extension PCR [43]. The correct insertion of the gene was confirmed by Sanger sequencing (Eurofin Genomics) and the generated plasmid termed pDAR15. For all cloning steps, *E. coli* strain NEB^®^ 5-alpha (NEB5α) (New England Biolabs Inc) was used. Plasmid amplification was done using QiaPrep Spin Miniprep Kit (Qiagen). Purified pDAR15 was stored at -20 °C. Transformed *E. coli* BL21 (DE3) was used for expression of WSCP1 (hereafter, WSCP).

### Production of WSCP

*E. coli* BL21 (DE3) containing the pDAR15 plasmid was taken from a 20% glycerol stock incubated overnight at 37 °C on LB agar containing 50 µg mL^−1^ ampicillin. A single colony was used to inoculate 20 mL of LB media containing 50 µg mL^−1^ ampicillin and incubated overnight at 37 °C and 200 rpm. Subsequently, 500 mL of LB, containing 50 µg ml^−1^ ampicillin, was inoculated to a final OD_600nm_ of 0.05 and incubated (37 °C; 200 rpm) until an OD_600nm_ of 0.5 was reached. The culture was then induced with 1 mM isopropyl-β-d-thiogalactopyranoside (IPTG) for 4 h. The cells were harvested by centrifugation for 15 min at 3000×*g* and pelleted cells were resuspended in 50 mL lysis buffer (50 mM NaH_2_PO_4_; 300 mM NaCl; 20 mM imidazole pH 7.8).

### Purification of WSCP

*E. coli* BL21 (DE3) cells were lysed using the CF1 Cell Disrupter (Constant Systems, Ltd.) at approximately 22 kPSI. The lysed sample was then centrifuged for 30 min at 4696×*g* to remove cell debris. WSCP was purified with two rounds of His-tag affinity chromatography. Briefly, the lysate was incubated for 1 h shaking (4  °C; 60 rpm) with Ni-NTA agarose beads. The beads were washed with 2 column volumes (CV) of each wash buffer containing increasing imidazole concentrations (50 mM NaH_2_PO_4_; 300 mM NaCl; 25, 50, and 60 mM imidazole, pH 7.8). WSCP was then eluted with 5 CV of elution buffer (50 mM NaH_2_PO_4_; 300 mM NaCl; 300 mM Imidazole). The eluted fractions were desalted using Amicon^®^ Ultra centrifugal filters with a 10 kDa cutoff (Merck Millipore). The protein samples were buffer exchanged with 50 mM sodium phosphate (pH 7.8). Protein concentration was determined with a Nanodrop spectrophotometer (Thermo Scientific) using the mass attenuation coefficient (E1%; 11.31 L g^−1^cm^−1^). This was calculated using the molar attenuation coefficient (ε_molar_; 28,420 M^−1^ cm^- 1^) and the molecular mass (*M*_r_; 25,122 Da). These values were calculated from the amino acid sequence of 6×His-WSCP using ExPASy ProtParam [44]. Protein purity and identity were verified by sodium dodecyl sulfate-polyacrylamide gel electrophoresis (SDS-PAGE) and immunoblotting, respectively.

### SDS-PAGE and immunoblotting

Protein samples were incubated at 95 °C for 5 min with SDS-Loading buffer (50 mM Tris-HCl, pH = 6.8, 10% glycerol, 2% SDS, 100 mM DTT, 0.05% bromophenol blue) and separated on a 12% Criterion™ XT bis-tris pre-cast protein gel (Bio-Rad). All gels were run in 2-morpholinoethanesulfonic acid (MES) running buffer (Bio-Rad) at 180 V. After SDS-PAGE separation, the gels were either stained with Coomassie or immunoblotted. For immunoblotting, the proteins were transferred to a 0.2 µm polyvinylidene difluoride (PVDF) membrane using a Trans-Blot^®^ Turbo Transfer System (Bio-Rad) for 7 min at 25 V. Afterwards the membranes were blocked for 1 h at RT with 5% skimmed milk powder (w/v) in phosphate buffered saline with 0.05% Tween-20 (PBS-T) buffer. The membrane was then incubated at 4 °C overnight with an anti-6X His primary antibody solution (1:100 dilution in PBS-T with 1% milk). The blot was then washed for 3 × 10 min with PBS-T and incubated with and anti-rabbit horseradish peroxidase-conjugated secondary antibody (1:5000) for 1 h at RT. The blot was rinsed (3 × 10 min with PBS-T) and developed using SuperSignal™ Chemiluminescent Substrate developer (Thermo Scientific) followed by immediate imaging (Additional file 1: Fig. S3).

### Chlorophyll extraction and purification

Chlorophyll *a* was extracted from the cyanobacterium *Synechococcus elongatus* UTEX 2973. The lyophilized cyanobacterial pellet was made into a fine powder using a pestle and mortar and resuspended in 100% methanol to extract pigments. The cell debris was spun down at 4000x*g* for 10 min at 4°C. The supernatant was removed using a MiniVac Evaporator (Labogene A/S, Denmark). Methanol extraction was repeated until the cyanobacterial pellet turned blue. The dried pigments were resuspended in 1:4 methanol:acetone and stored at − 20 °C.

Thin Layer Chromatography (TLC) was used to separate the pigments on RP-18 F_245_s silica gels. The TLC mobile phase was comprised of 7:11:1 acetone:methanol:ddH_2_O mixture. A dark green band containing chlorophyll *a* (Additional file 1: Fig. S4) was scraped off and dissolved in 100% acetone. The silica was then spun down at 5000×*g* and the supernatant was removed and evaporated using a Spin-Vac. After removing the acetone, the Chl *a* was resuspended in 96% ethanol (EtOH) and stored in the dark at − 20 °C. The amount of Chl *a* was calculated using the ε_molar_ of 74,400 cm^−1^ M^−1^ [26].

### WSCP–Chl *a* reconstitution

Reconstitution was performed with a 5× molar excess of Chl *a* to WSCP for a fully saturated complex (i.e., 4Chl*a*:4WSCP) according to Palm et al. [26]. In short, Chl *a* was solubilized in a 96% EtOH solution which was added dropwise to the WSCP protein solution to a final concentration of 20% EtOH to avoid protein denaturation. The mixture was incubated at RT for 30 min at 1000 rpm in the dark.

The reconstitution mix was then buffer exchanged with 50 mM sodium phosphate buffer (pH 6.3) to remove excess EtOH with 30 kDa cutoff Amicon^®^ Ultra centrifugal filters (Merck Millipore). The reconstitution mix was placed in a new microcentrifuge tube which was then centrifuged at 15,000×*g* for 10 min. The supernatant was removed and stored at 4 °C in the dark. Stoichiometry of the reconstituted complex was determined by measuring Chl *a* (673 nm) and WSCP (280 nm) absorbance using 10 mm quartz absorption cuvettes. Absorption spectra were measured between 250 and 750 nm and compared with published data (Additional file 1: Fig. S5) [26].

### Assay for determination of photostability

Chl *a* and WSCP–Chl *a* fluorescence was compared under various conditions using the Biotek Synergy™ microplate reader with Gen5™ Data Analysis Software. The fluorescence was measured at 420 nm and emission was integrated over 650–700 nm. Assays had a total volume of 200 µL and were performed in black 96-well Nunc™ optical plates (Thermo Scientific). Assay mix included *Tt*AA9 (0.035 mg mL^−1^), ascorbic acid (1 mM) and Chl *a* or WSCP–Chl *a* (2.6 µM) (OD = 0.2, ε_molar_ of 74,400 cm^−1^ M^−1^) calculated according to Palm et al. [26] and 50 mM sodium phosphate buffer (pH 6.3). Varying conditions included light intensity (50, 200, 500 µmol m^−2^ s^−1^), temperature (25 and 50 °C) and pH (50 mM potassium phosphate buffer pH 5, 6, 7, 8). Illumination was with cool white LEDs (4000 K spectrum) in a customized light rig and powered by Velleman™ DC Lab Switching Mode Power Supply. All experiments were performed in triplicates and data shown are the averages with the standard error of the mean (SEM).

### Assays for the determination of H_2_O_2_ production

The production of H_2_O_2_ in the light-driven assays was measured using Ampliflu™ Red (Sigma) according to Singh et al. [45]. All reactions were performed in black 96-well Nunc™ optical plates (Thermo Scientific). The assay was sampled every 10 min and 10 µL samples were mixed with 2 µL Ampliflu™ Red (5 mM stock), 15 µL Horseradish Peroxidase from Sigma (300 U mL^−1^), 2 µL Ethylenediaminetetraacetic acid (EDTA) (10 mM stock), and 171 µL 50 mM K_2_PO_4_ (pH 6.0). Measurements were made on Biotek Synergy™ microplate reader preheated to 37 °C and analyzed with Gen5™ Data Analysis Software. Excitation was set to 557 nm and emission was measured at 583 nm with three replicate reads per reaction. A standard curve was prepared from ≥ 30% H_2_O_2_ for trace analysis from Sigma Aldrich. The assays were composed of varying conditions included light intensity (0, 50, 100, 200, 500 µmol m^−2^ s^−1^) and ascorbic acid (0, 250, 500, 1000 µM). All experiments were performed in triplicates and data shown are the averages with the standard error of the mean (SEM).

### Light-driven assays

Productivity assays were performed using *Tt*AA9 from Novozymes A/S. Purification and copper loading of *Tt*AA9 was performed according to Singh et al. [45]. From a stock solution of 7 mg mL^−1^, a final concentration of 0.035 mg mL^−1^ *Tt*AA9 was used in each assay. The substrate stock was 0.5% w/v cellulose nanofibrils (CNF) with a final concentration of 0.25% w/v used in all assays. 50 mM sodium phosphate (pH 6.3) was used in all assays and the reductant ascorbic acid (Asc) was purchased from Sigma Aldrich and aliquoted in 200 mM stock solutions and kept at − 20 °C. The detergent, n-dodecyl-β-d-Maltoside (β-DM) was kept at − 20 °C in 2% stock solutions and 0.03% in assays. This was used to keep Chl *a* soluble for control assays. Assays were carried out at either 25 or 50 °C using an Eppendorf Thermomixer while mixing at 1000 rpm. Samples were filtered using 0.22 µm MilliporeSigma™ MultiScreen_HTS_ Durapore™ 96-well plates (Fischer Scientific) immediately after the assay completion. This removes the substrate from the reaction, stopping further catalysis by *Tt*AA9.

### Gluconic acid determination

The soluble fraction of CNF treated with *Tt*AA9 was incubated overnight at 40 °C with 8 µg mL^−1^ β-glucosidase from *Aspergillus niger* (Megazyme Cat. No. E-BGLUC w/ 50 U mg^−1^). This leads to the hydrolysis of terminal, non-reducing β-d-glucosyl residues with release of β-d-glucose and gluconic acid (C1-oxidation product) [46]. Gluconic acid was then determined using the d-Gluconic Acid/d-Glucono-δ-lactone Assay Kit from Megazyme, following the manufacturer’s instructions for a microplate assay. Absorption was measured using Biotek Synergy™ microplate reader at 340 nm. All experiments were performed in triplicates and data shown are the averages with the SEM.

### Oligosaccharide production analyzed by HPAEC–PAD

High-Performance Anion Exchange Chromatography (HPAEC) was used to analyze released oligosaccharides after LPMO-driven cellulose oxidation. HPAEC was performed on Dionex™ ICS-5000+ with a PAD detector from Thermo Scientific. A CarbonPac PA1 column (two 2 × 50 mm guard columns followed by a 2 × 250 mm analytical column) was run with a flow rate of 0.25 mL min^−1^ at 30 °C. The aldonic acids were separated chromatographically as previously described [47]. The elution gradient was (Eluent A: 0.1 M NaOH; Eluent B: 1 M NaOAc in 0.1 M NaOH): 100% A:0% B to 90% A:10% B (10 min), then to 83.1% A:16.9% B (25 min) and lastly 0% A:100% B (30 min). For reconditioning of the column 100% A:0% B was applied for 15 min (35–50 min). The C1-oxidized oligosaccharides were assigned based on standards from previous studies [8, 48]. Curves are the average of triplicate experiments.

## Supplementary information

**Additional file 1.** Additional figures.

## Data Availability

All appropriate data for the study has been included in the manuscript.

## Abbreviations

Asc: Ascorbic acid
β-DM: β-d-Maltoside
Chl: Chlorophyll
CNF: Cellulose nanofibrils
CV: Column volume
EtOH: Ethanol
HPAEC: High-performance anion exchange chromatography
H_2_O_2_: Hydrogen peroxide
LPMO: Lytic polysaccharide monooxygenase
O_2_: Oxygen
^1^O_2_: Singlet oxygen
O_2_^−^: Superoxide
SDS-PAGE: Sodium dodecyl sulfate-polyacrylamide gel electrophoresis
SEM: Standard error of the mean
SOSG: Singlet oxygen sensor green
*Tt*AA9: LPMO from *Thielavia terrestris*
WSCP: Water-soluble chlorophyll protein
