# Supplementary material for: Water-soluble chlorophyll-binding proteins from Brassica oleracea allow for stable photobiocatalytic oxidation of cellulose by a lytic polysaccharide monooxygenase
Source: Biotechnol Biofuels. 2020 Nov 30;13:192. doi: 10.1186/s13068-020-01832-7 (PMC7708235; doi:10.1186/s13068-020-01832-7)
Supplement: Supplementary file 1 — Additional file 1. Additional figures. [file 13068_2020_1832_MOESM1_ESM.docx]

**Additional Material for**

**“Water-soluble chlorophyll-binding proteins from *Brassica oleracea* allow for stable photobiocatalytic oxidation of cellulose by a lytic polysaccharide monooxygenase**.”

N. Dodge^1^, D. A. Russo^2^, B.M. Blossom^3^, R.K. Singh^4^, B. van Oort^5^, R. Croce^5^, M. J. Bjerrum^4^, P. E. Jensen^1^

^1^Department of Food Science, University of Copenhagen, Rolighedsvej 26, DK-1958 Frederiksberg

^2^Department of Bioorganic Analytics, Institute for Inorganic and Analytical Chemistry, Friedrich Schiller University Jena, Jena, Germany

^3^Department of Geosciences and Natural Resource Management, University of Copenhagen, Frederiksberg C, Denmark

^4^Department of Chemistry, University of Copenhagen, Copenhagen, Denmark

^5^Biophysics of Photosynthesis, Department of Physics and Astronomy, Faculty of Sciences, and LaserLaB Amsterdam, Vrije Universiteit Amsterdam, Amsterdam, Netherlands


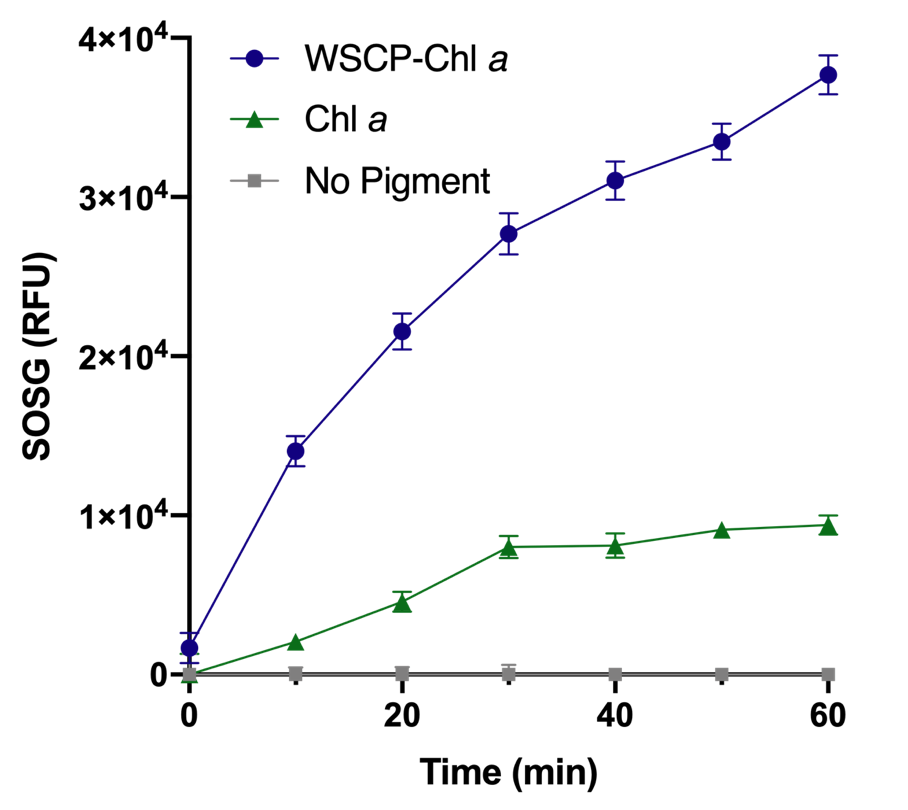


**Figure S1. Apparent singlet oxygen (^1^O_2_) measurements using Singlet Oxygen Sensor Green™ (SOSG).** SOSG was purchased from Invitrogen™ and used according to Agostini et al. 2017. The assay was performed by combining 2 µM SOSG with Chl *a* or WSCP-Chl *a* (2.6 µM) and 50 mM sodium phosphate buffer (pH 5.0) with 2% methanol (v/v). SOSG emission was measured in the Biotek Synergy™ (Ex: 480 nm, Em: 530 nm) every 10 mins. The assay was performed at 50 °C for a total of 60 min in 50 µmol m^-2^ s^-1^. The average of three independent experiments and SEM is plotted.

**
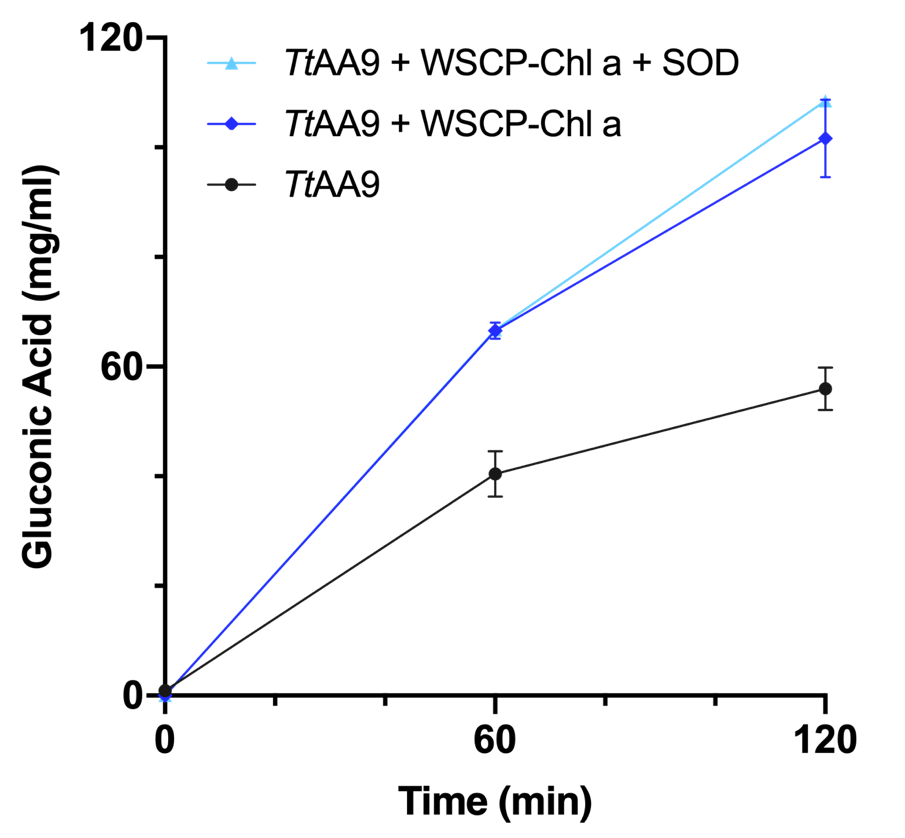
**

**Figure S2. Gluconic acid assay with superoxide dismutase (SOD).** SOD was used in the assay in order to determine the involvement of superoxide (O_2_^-^). The assay was performed at 50 µmol m^-2^ s^-1^ cool white LED (4000K) spectrum at 50°C in 50 mM potassium phosphate buffer (pH 6), with CNF (0.25 % w/v) and either *Tt*AA9 without pigment, *Tt*AA9 and WSCP-Chl *a*, or *Tt*AA9 and WSCP-Chl *a* with SOD (100 µg ml^-1^). Asc feed rates were 500 µM/hr. The averages of three independent experiments and the SEM of these experiments is shown. A t-test was done on the final gluconic acid concentration (120 min) between ‘*Tt*AA9 + WSCP-Chl ‘*a* and ‘*Tt*AA9 + WSCP-Chl *a* + SOD’ to demonstrate the effect of SOD. There was no significant difference (p > 0.05).


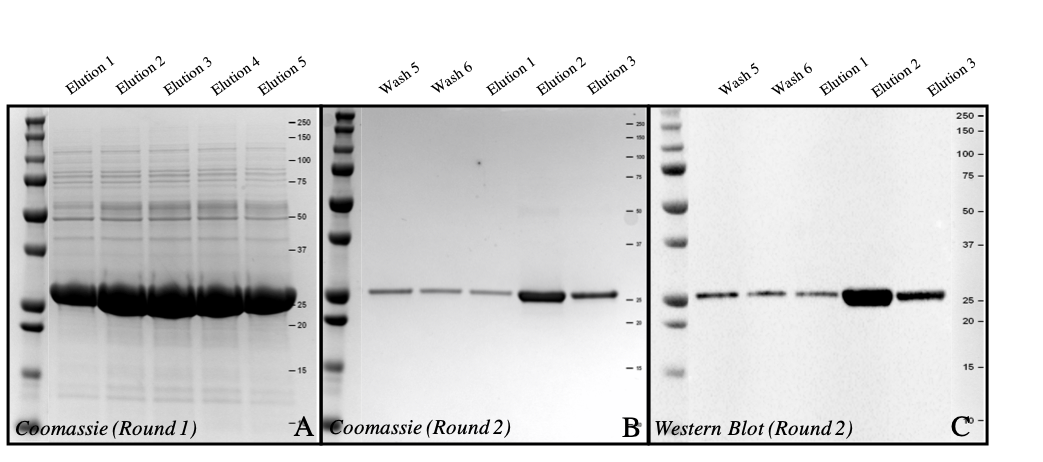


**Figure S3. Gel images of WSCP** (25.12 kDa) after purification (A) Coomassie image of elutions from 1^st^ round of His-Tag purification. (B) Coomassie of elutions from 2^nd^ round of His-Tag purification. (C) Western Blot of second round of His-Tag purification.


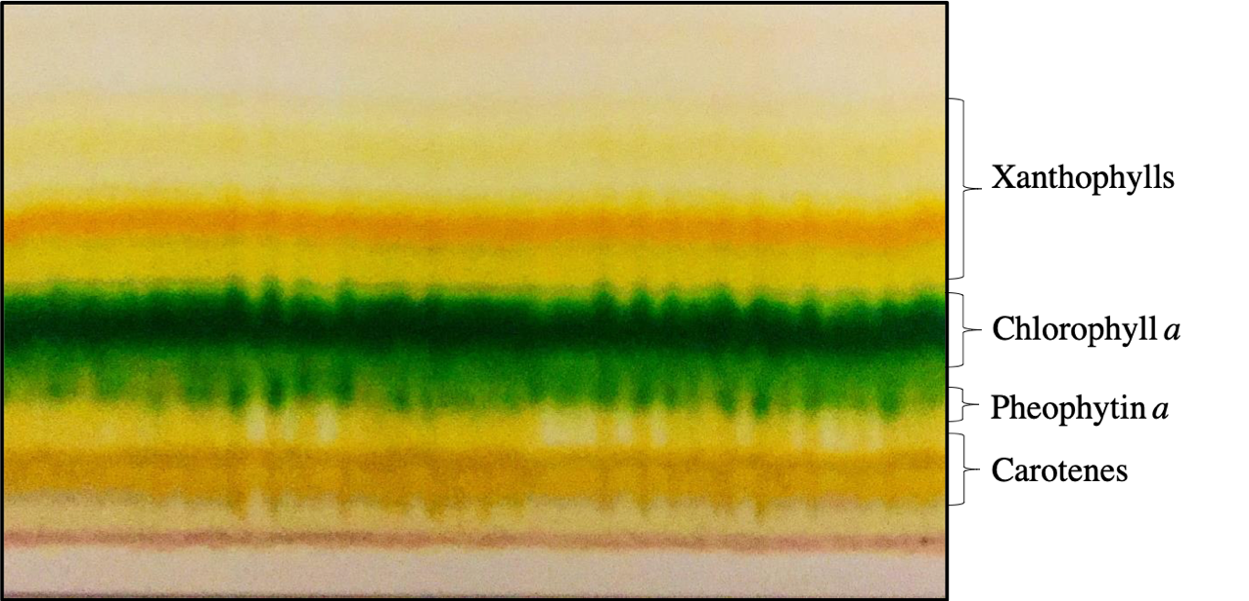


**Figure S4. TLC of pigments extracted from *Synechococcus elongatus* UTEX 2973.** Separated by reverse phase TLC, visible bands starting from the bottom in order of polarity correspond to carotenes, pheophytin *a*, chlorophyll *a*, and xanthophylls.


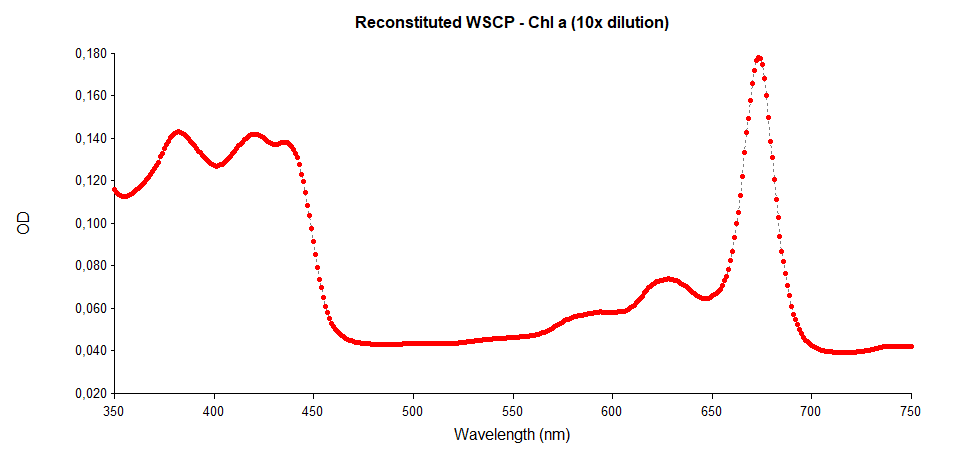


**Figure S5. Absorbance spectrum from 250-750 nm of reconstituted WSCP-Chl *a***. Maximum peak (Q_y_) is seen at 673 nm.


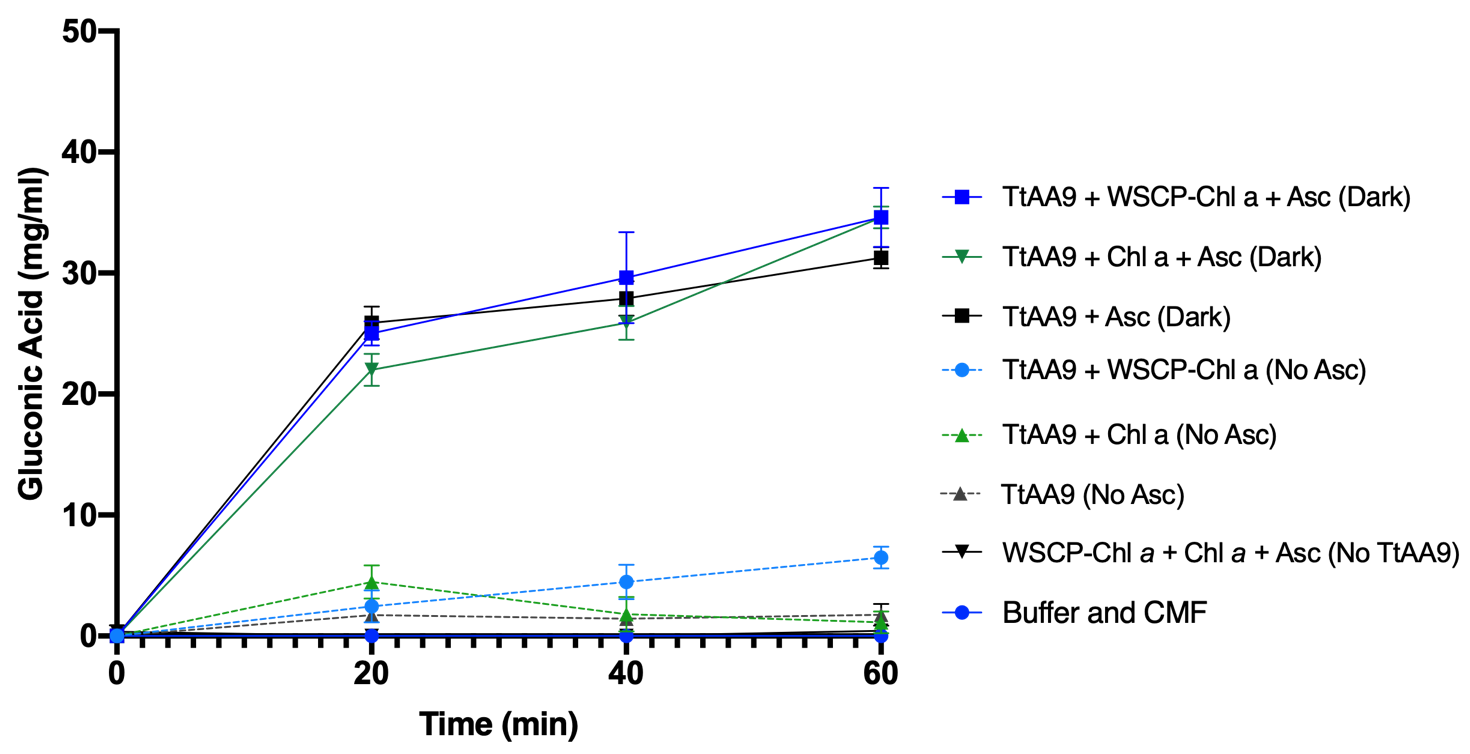


**Figure S6. Gluconic acid assay with control experiments.** Light experiments were performed at 100 µmol m^-2^ s^-1^ cool white LED (4000K) spectrum at 25°C in 50 mM potassium phosphate buffer (pH 6.3), with CNF (0.25 % w/v). Dark controls were covered with aluminum foil. 1 mM Asc was added at time 0 hour. Curves are averages of three independent experiments and the SEM of these experiments is shown. Single factor ANOVA was performed on the dark controls and there is no significant difference upon the addition of pigments without additional light (p > 0.05).
